# Isolation and characterization of vesicular and non-vesicular microRNAs circulating in sera of partially hepatectomized rats

**DOI:** 10.1038/srep31869

**Published:** 2016-08-18

**Authors:** Mirco Castoldi, Claus Kordes, Iris Sawitza, Dieter Häussinger

**Affiliations:** 1 Department of Gastroenterology, Hepatology and Infectious Diseases, Heinrich Heine University, Düsseldorf, D40225, Germany

## Abstract

Circulating microRNAs are protected from degradation by their association with either vesicles or components of the RNAi machinery. Although increasing evidence indicates that cell-free microRNAs are transported in body fluids by different types of vesicles, current research mainly focuses on the characterization of exosome-associated microRNAs. However, as isolation and characterization of exosomes is challenging, it is yet unclear whether exosomes or other vesicular elements circulating in serum are the most reliable source for discovering disease-associated biomarkers. In this study, circulating microRNAs associated to the vesicular and non-vesicular fraction of sera isolated from partially hepatectomized rats were measured. Here we show that independently from their origin, levels of miR-122, miR-192, miR-194 and Let-7a are up-regulated two days after partial hepatectomy. The inflammation-associated miR-150 and miR-155 are up-regulated in the vesicular-fraction only, while the regeneration-associated miR-21 and miR-33 are up-regulated in the vesicular- and down-regulated in the non-vesicular fraction. Our study shows for the first time the modulation of non-vesicular microRNAs in animals recovering from partial hepatectomy, suggesting that, in the search for novel disease-associated biomarkers, the profiling of either vesicular or non-vesicular microRNAs may be more relevant than the analysis of microRNAs isolated from unfractionated serum.

Since the discovery that microRNAs (miRNAs) are present in body fluids in a cell-free form, the analysis of circulating nucleic acids has developed into a mainstream research topic^1^. Reason for this is that the profiling of cell-free miRNAs has shown potentials in the classification of patients with several diseases including cardiovascular diseases^2^,^3^, coronary artery disease^4^, chronic kidney disease^5^, alcoholic liver disease^6^ and viral hepatitis^7^,^8^. However, as the analysis of circulating miRNAs is still in its infancy, it suffers from a lack of standardized procedures at both pre-analytical and analytical level and as result of these deficits the comparison between different studies is challenging^9^. An unavoidable layer of variability is generated by the intrinsic nature of cell-free miRNAs. Indeed, miRNAs circulating in body fluids are transported by heterogeneous carriers including extracellular vesicles (i.e., exosome, 30–100 nm^10^,^11^), microparticles (50 nm–1 μm^12^,^13^) and apoptotic blebs (100 nm–5 μm^14^,^15^); for a review see ref. 16], high and low density lipoproteins^17^ as well as in a vesicle-free form associated to RNA-binding proteins^18^,^19^,^20^. Unfortunately, as sizes and properties of different types of extracellular vesicles overlap considerably, it is not possible to efficiently purify a specific type of vesicle (see for a detailed review see ref. 21). To date exosomes are the most commonly investigated extracellular vesicles. Exosomes are vesicles of heterogeneous size and composition delimitated by a lipid bilayer of endosomal origin^10^,^11^. What makes exosomes so attractive to study is that the selective transfer of cargoes via the exosomal pathway is emerging as an essential path for intercellular communication in both, health and disease^22^. Remarkably, transportation and delivery of functional molecules is not unique to exosomes, but it is emerging as a common feature of extracellular vesicles such as microparticles^13^, apoptotic blebs^14^,^15^, lipoproteins^17^ which all have been reported to transport and functionally deliver miRNAs to target cells. To date no functional relevance has been associated to miRNAs circulating in blood associated to proteins. These non-vesicular miRNAs, which are thought to originate from the release of cellular contents into the extracellular space following lytic events^23^, escape RNase activity by tightly interacting with proteins such as RNA binding proteins [i.e., Argonaute2 (Ago2)^18^ and Argonaute1 (Ago1)^19^] or nucleoplasmin-1 (NPM1)^20^.

The aim of the present study was to evaluate to which extent levels of vesicular and non-vesicular miRNAs are modulated in sera of animals recovering from partial hepatectomy (PHx). To circumvent obvious limitations associated to ‘standard’ protocols for vesicle enrichment (i.e., long preparative intervals and low throughput sample number), nanoparticle-tracking analysis (NTA) was used to measure and characterize the size and distribution of vesicles isolated under different conditions^24^. NTA is a novel approach that allows to measure both, distribution and size of particles by means of video tracking. Specifically, laser scattering microscopy is used to track random movements of vesicles in solution resulting from Brownian motions enabling the visualization of particles as small as 20 nm. Here we show that isolation of vesicles through ultracentrifugation only pellets a fraction of the vesicles present in rat serum, hence resulting in a significant underestimation of vesicles number, which possibly underrates the contribution of the vesicle-associated cargo to inter-cellular communication. We also show that the size of vesicles circulating in ‘naïve’ rat serum range between 100 and 150 nm, therefore providing evidence that the procedure of filtering serum for small vesicles enrichment (i.e., as carried out during isolation of exosomes) is not required. Furthermore, we determined that miRNAs circulating in rat serum, similarly to human plasma^18^, can be assigned to three distinct groups. One group, which was found enriched in vesicle-depleted sera, another which was found enriched in RNA isolated from vesicles-enriched pellets, while a third group of miRNAs was found evenly distributed between the two different fractions of the serum.

This acquired knowledge was used to measure the levels of vesicle- and protein-associated miRNAs circulating in sera of rats after partial hepatectomy. We found that miR-21 and miR-33, which are known as regeneration-associated miRNAs, were up-regulated in the vesicular and down-regulated in the non-vesicular fraction of the serum, while expression levels of inflammation-associated miR-150 and miR-155 were significantly increased in the vesicular fraction only. Finally, a third group of miRNAs, comprising Let-7a and the liver enriched miR-122, miR-192 and miR-194, were found to change in parallel in both fractions. Analysis of cellular and extracellular vesicles (EVs)-associated miRNAs in cultured rat hepatic stellate cells (HSCs) stimulated with cytokines [such as Interleukin-6 (IL-6) or transforming growth factor-beta1 (TGF-β1)] identifies a widespread modulation of miRNAs expression and secretion. These data suggest that the pleiotropic activity of disease-associated cytokines on both cellular and EVs-associated miRNAs may affect hepatic functionality in response to injury and regeneration. However, further studies are required to assess whether the panel of miRNAs studied here is prognostically or diagnostically relevant in patients with liver disease. Overall, the present findings indicate that the analysis of miRNAs needs to address both, the vesicular and the non-vesicular fraction of the serum, when compared to the analysis of the total (i.e., unfractionated) population of circulating miRNAs.

## Results

### Nanoparticle-tracking analysis enables the quantification of circulating extracellular vesicles

**Figure 1:**
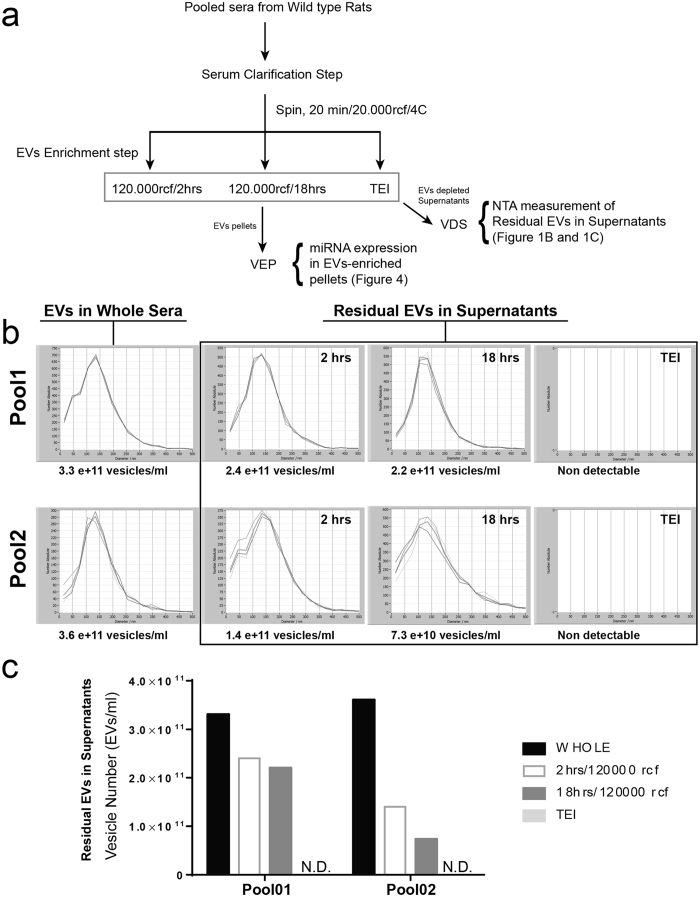
TEI precipitation recovers extracellular vesicles more efficiently as compared to ultracentrifugation. (**a**) Schematic representation of experimental procedure is shown. Vesicles were isolated from independent pools of rat sera by either addition of TEI or by ultracentrifugation for 2 (standard) or 18 (extended) hours. Following NTA was used to measure residual vesicles in the supernatant, compared to the number of vesicles present in the whole sera (Not Centrifuged). Measurements from two independent pools are shown. (**b**) Residual EVs distribution and (**c**) Number of residual EVs as measured by NTA. N.D. Non-detectable.

**Figure 2:**
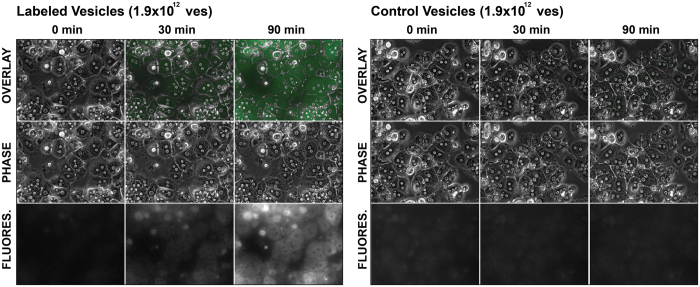
Vesicles isolated from rat sera via TEI-precipitation are up taken by rat primary hepatocytes. To assess whether the vesicles isolated via TEI-induced precipitation were active, freshly isolated rat primary were incubated over night with 1.9 × 10^12^ vesicles, which were either stained with Syto RNAselect (left panel) or left unstained (right panel). Vesicle uptake was recorded by live imaging.

NTA is an emerging technology for visualization, measurement and quantification of extracellular vesicles. In this study, NTA was used to compare the efficiency in vesicles recovery between differential centrifugation and polymer-based precipitation (Total Exosome Isolation reagent; TEI). Isolation of vesicles starting from serum is commonly carried out by using differential centrifugation. Through this approach samples are subjected to stepwise increased centrifugal force to first remove unwanted particles (i.e., cellular debris) and then for pelleting vesicles of desired size. On the other hand TEI-based precipitation enables the pelleting of vesicles at low centrifugal force (i.e., ~10 to 20000 rcf). Importantly, through the NTA analysis of the vesicle-depleted sera we found that the supernatant of sera subjected to ultracentrifugation contains significant amounts of EVs, while no EVs were detected in the supernatant of sera subjected to TEI precipitation (Fig. 1). Therefore, TEI-based precipitation recovers more vesicles compared to both standard (2 hrs, 120000 rcf) and extended (18 hrs, 120000 rcf) ultracentrifugation. Moreover, vesicles precipitated by using TEI are functional as they are internalized by primary hepatocytes in culture (Fig. 2). As already reported by other studies^21^, we also noticed that ultracentrifugation recovers vesicles with variable efficiency. Interestingly, this seems to be directly proportional to the viscosity of the solution. Despite the differences in the number of recovered vesicles between TEI-based precipitation and ultracentrifugation (Fig. 1c), no significant differences in the size distribution of vesicles were observed (Fig. 1b). Hence, TEI precipitation is a fast, efficient and inexpensive alternative to ultracentrifugation for recovering vesicles circulating in rat serum.

### Effect of sample pre-clearing on EVs distribution

**Figure 3:**
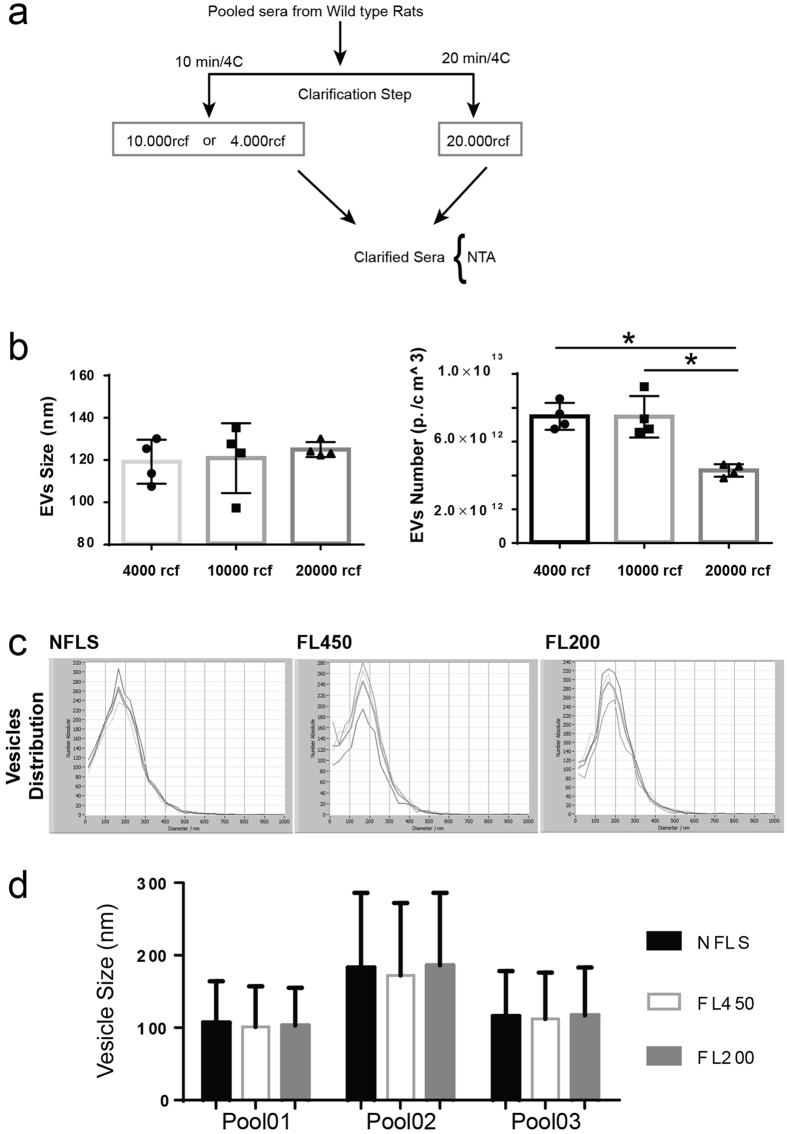
Filtration does not affect distribution of vesicles isolated by TEI-induced precipitation. Pool of rat sera were either filtered through 0.2 μm (FL200) or 0.45 μm (FL450) syringe filters or left unfiltered (NFLS) before isolation by addition of appropriate quantity of TEI. Following, vesicle distribution was measure by using NTA. (**a**) Schematic representation of experimental procedure is shown. (**b**) Vesicles size distribution and (**c**) vesicles peak as measured by NTA for three independent pools. Data are represented as average ± standard deviation. *P ≤ 0.05.

A step common to a number of published method for isolating EVs is to carry out a pre-clearing step to remove cellular debris and other insoluble particles. However, a wide range of centrifugation speed for this step can be found. To assess the effect of centrifugal forces on EVs distribution, the scheme depicted in Fig. 3a was applied. To minimize experimental variability, sera isolated from wild type animals were pooled and identical aliquots were centrifuged at either 4000 or 10000 rcf (10 min.) or 20000 rcf (20 min.). Following, vesicles contained in cleared supernatants were analyzed by NTA (Fig. 3b). A significant reduction in the number of EVs was observed in the sample subjected to the highest centrifugal force (i.e., 20000 rcf). However, EVs distribution was not affected by the pre-clarification step. Importantly, it was found that samples centrifuged at 20000 rcf displayed a smaller variance. Therefore, this centrifugation speed served as standard speed for carrying out pre-clarification of the samples. Mechanical filtration through filters with a pore size of 200 nm is a method frequently found in publications to enrich biological fluids of small size vesicles (i.e., <200 nm). To evaluate to which extent filtration affects the distribution (or the number) of vesicles, independent pools of rat sera were subjected to filtration through either 450 nm (FL450) or 200 nm (FL200) syringe filters or left un-filtered (NFLS). Following, equal volumes of NFLS, FL450 and FL200 sera were subjected to TEI precipitation and vesicles distribution were measured by NTA. Data analysis indicates that no significant differences in either vesicles size or distribution (Fig. 3c). Specifically, we found that filtration through either 200 or 450 nm filters does not affect vesicles distribution [Fig. 3c, Pool01; (NFLS 107 ± 57 nm, FL450 101 ± 56 nm and FL200 103 ± 52 nm), Pool02; (NFLS 183 ± 103 nm, FL450 172 ± 100 nm and FL200 186 ± 100 nm) and Pool3; (NFLS 116 ± 62 nm, FL450 112 ± 64 nm and FL200 117 ± 66 nm)]. It also had no effect on the peak of the distribution [Fig. 3d, Pool01; (NFLS 133,97 nm, FL450 127,43 nm and FL200 138,63 nm), Pool02; (NFLS 167,73 nm, FL450 163,27 nm and FL200 175,80 nm), and Pool3; (NFLS 129,00 nm, FL450 123,10 nm and FL200 128,25 nm)]. Overall, the presented data indicate that filtration is dispensable for enriching rat sera in small size vesicles.

### Ultracentrifugation recovers lower amounts of miRNAs compared to TEI-based precipitation

**Figure 4:**
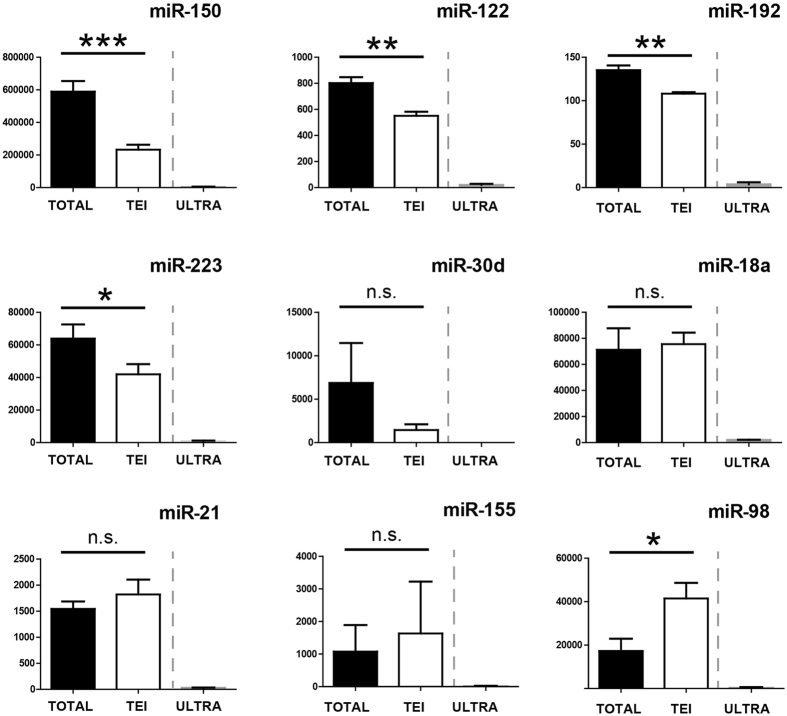
Expression of VEP-associate miRNAs isolated from rats sera by using Ultracentrifugation and TEI. RNAs isolated from whole sera (TOTAL), from vesicles pelleted by either Total Exosome Isolation reagent-based (TEI) or Standard ultracentrifugation (ULTRA) were reverse transcribed following the miQPCR protocol, and the expression of a panel of miRNAs was analyzed by individual qPCR assays. qPCR data were median normalized as described in ref. 52. Data are represented as average ± standard deviation calculated from four independent isolations (n = 4). *P ≤ 0.05; **P ≤ 0.01; ***P ≤ 0.0001; n.s., non-significant.

Deranged levels of cell-free circulating miRNAs have been linked to the diagnosis and prognosis of human diseases^2^,^3^,^4^,^5^,^6^,^7^,^8^. To date differential ultracentrifugation is the ‘gold’ standard for isolating EVs and for quantification of vesicle-associated miRNAs. Using qPCR we evaluated whether the lower number of vesicles isolated via ultracentrifugation correlated with a lower miRNA count. For this purpose, 200 μl of pooled-rat sera, or an equivalent volume of PBS as negative control, were either directly dissolved in phenol for RNA isolation or subjected to TEI precipitation. For vesicles isolation by ultracentrifugation, 10 ml of pooled rat sera were centrifuged at high speed (2 hours, 120000 rcf) and pelleted vesicles were diluted into an appropriate amount of PBS. Following, a volume equivalent to 200 μl of the original volume was used for RNA extraction. Determination of RNA concentration by using either photometric (i.e., Nanodrop) or fluorimetric (i.e., Qubit) methods did not show any measurable yield (data not shown). This is in line with reports from other research groups, which determined that the concentration of cell-free RNA per milliliter of blood is in the range of 1 to 150 ng^25^,^26^. Hence, the amount of RNA that can be recovered from 200 μl of rat serum is below the detection limit of standard techniques for RNA quantification. Therefore, the amount of material to be used for cDNA synthesis was calculated on volume of elute material as previously described^27^. We found that signals generated by RNAs isolated from either the total serum (TOTAL) or TEI-precipitated vesicles were comparable, whereas signals generated by RNAs isolated from vesicles pelleted via ultracentrifugation (ULTRA) were significantly lower (Fig. 4). Based on these data we concluded that the lower number of vesicles recovered via ultracentrifugation negatively affect miRNA quantification by using qPCR. Composition between the TOTAL and the PEI shows that signals generated by four miRNAs (i.e., miR-150, -122, -192 and -223) were significantly higher in the TOTAL compared to the TEI cDNAs. This data indicates either that a predominant proportion of these miRNAs is associated with the non-vesicular fraction of the serum or that the RNA extraction method used in this study recovers these miRNAs with higher efficiency when starting from the whole serum. Interestingly, miR-98 was found significantly enriched in the TEI compared to TOTAL RNAs. Overall, we concluded that the RNA extraction method used in this study might recover miR-98 less efficiently when starting from whole sera compared to vesicle-enriched pellets. Finally, no significant differences were detected between the TOTAL and TEI RNAs fractions for the remaining four miRNAs included in this panel (i.e., miR-30d, -18, -21 and -155). This data suggests that these miRNAs are predominantly associated with the vesicular fraction of the serum.

### Filtration does not influence the amount of miRNAs in RNAs isolated from vesicle- enriched pellets and vesicle-depleted serum

**Figure 5:**
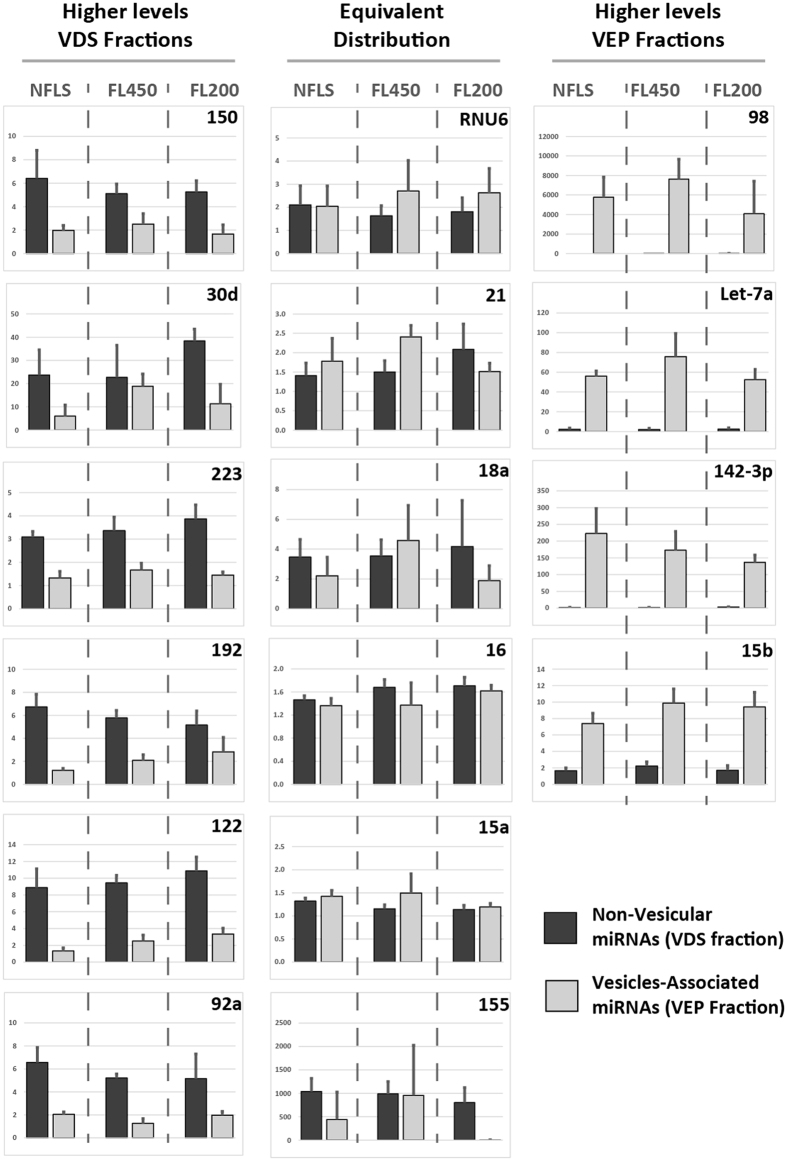
Effect of filtration on expression profiling of VEP- and VDS-associated miRNAs. Vesicular and non-vesicular RNAs were extracted from sera isolated from wild type rats. Expression of miRNAs present in the VDS and VEP fractions isolated from (**a**) non filtered (NFLS) serum, (**b**) serum filtered through 0.2 μm (FL200) or (**c**) serum filtered through 0.45 μm (FL450) were measured by using miQPCR. Data are represented as average ± standard deviation from 4 independent isolations (n = 4).

In Fig. 3 we show that filtration of rat sera affects the number but not the size distribution of the isolated vesicles as measured by NTA. We next evaluated whether filtration affects the expression level of vesicle- and protein-associated miRNAs by using qPCR. For this purpose, identical aliquots of pooled rat sera were either filtered [450 nm (FL450) or 200 nm (FL200)] or left un-filtered (NFLS), vesicles were separated from sera with TEI precipitation, RNAs contained in vesicle-depleted sera (VDS) and vesicle-enriched pellets (VEP) were isolated by phenol chloroform. Next, expression levels of selected miRNAs were measured in the different samples by using miQPCR^27^ (Fig. 5). Similarly, with the data presented in Fig. 4, three different groups of miRNAs were found in rat serum. A group of miRNAs whose expression is higher in the vesicle-depleted RNAs (i.e., miR-150, -30d, -122, -92a, -223 and -192, Fig. 5, left column). A second a group of miRNAs whose expression is higher in the vesicles-enriched pellets (i.e., Let-7a, miR-15b, -142-3p and -98, Fig. 5, right column) and a third group of miRNAs which presents comparable expressions in both groups (i.e., miR-15a, -16, -155, -21, -18a and RNU6, Fig. 5, middle column). Significantly, in a previously published study from Arroyo *et al*.^18^ Let-7a and miR-98 were found enriched in the vesicle-enriched and in the vesicle-depleted fractions of the serum respectively. Furthermore, the presented data indicate that filtration does not significantly affect the levels of miRNAs associated with either the vesicular (VEP) or the non-vesicular (VDS) fraction of rat serum.

### Modulation of vesicular- and non-vesicular miRNAs circulating in the serum of partially hepatectomized rats

The knowledge acquired throughout this study on the size, distribution, as well as behavior upon pre-clearance and filtration of vesicular- and non-vesicular miRNAs was applied to measuring the levels of cell-free miRNAs circulating in sera of rats recovering from partial hepatectomy. Importantly, this experimental model was chosen, because liver regeneration under these conditions is a frequently studied and well-characterized model of tissue regeneration in mammals, and because body’s response to PHx is expected to modulate the expression of cell-free circulating miRNAs^28^,^29^,^30^. For this purpose, vesicles were isolated from sera prepared from the blood of control (operated) animals (day 0; n = 3) as well as from the blood of rats recovering from partial hepatectomy (2, 4 and 6 days after PHx; n = 3) by using TEI precipitation. Thereafter, RNAs were isolated from both the vesicle-enriched pellets and vesicles-depleted sera and the expression of selected miRNAs was analyzed by miQPCR^27^.

### Analysis of vesicle-associated miRNAs following PHx identifies the modulation of liver-enriched miRNAs as well as of miRNAs associated with regeneration and inflammation

**Figure 6:**
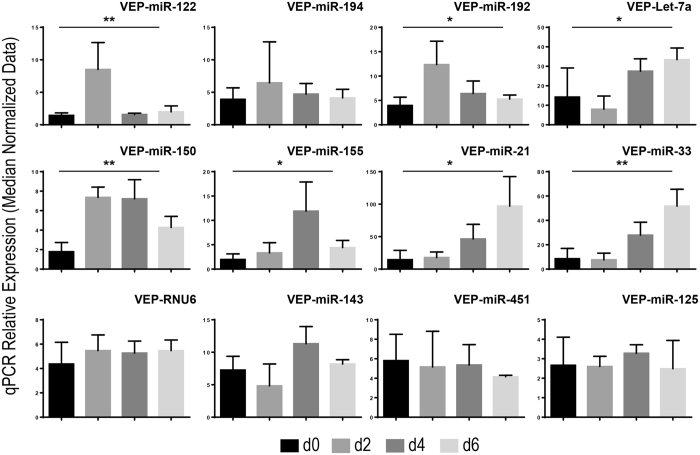
Expression of vesicles-associated miRNAs isolated from the serum of rats after partial hepatectomy. Vesicles were isolated via TEI precipitation from sera of rats recovering from PHx and RNAs were isolated from VEP fractions of sera. qPCR data were median normalized as described in ref. 52. Data are represented as average ± standard deviation. Statistical analysis was performed by One-Way Anova (day 0; n = 3) versus individual groups (day 2, 4 and 6; n = 3 within each group) for each miRNA. *P ≤ 0.05; **P ≤ 0.01.

Expression levels of vesicle-associated miRNAs contained in the serum of rats at day 0 (i.e., before PHx), 2, 4, and 6 days after PHx were measured by using miQPCR (Fig. 6). The analysis shows that liver-enriched miRNAs [miR-122 (P = 0.007) and miR-192 (P = 0.03)] are up-regulated at day 2 after PHx and the levels are reduced to almost pre-PHx levels (i.e., day 0) at days 4 and 6 after PHx (Fig. 6, top row). Interestingly, also the ubiquitously expressed miRNA Let-7a (P = 0.03) is significantly up-regulated in the serum of these animals, but with different dynamics compared to liver-enriched miRNAs (Fig. 6, top row). The expression of miRNA associated with infection and inflammation [i.e., miR-150 (P = 0.01) and miR-155 (P = 0.04)] is up-regulated through day 2 and 4, and declines at day 6 (Fig. 6, middle row). The expression of miRNA associated to regeneration [i.e., miR-21 (P = 0.03) and miR-33 (P = 0.04)] is up-regulated over the analyzed time points reaching the highest-level 6 days after PHx (Fig. 6, middle row). Importantly, the expression of hematopoiesis-associated miRNA (miR-451), brain-enriched miRNA (miR-143), hepatoblastoma-associated miRNA (miR-125) and of the short non-coding RNA RNU6 remained unchanged during the observed period (Fig. 6, lower row), indicating that the amount of these vesicle-associated miRNAs is not modulated at 2, 4 and 6 days after PHx.

### The expression of miRNAs associated to the non-vesicular fraction of the serum is modulated in rats recovering from PHx

**Figure 7:**
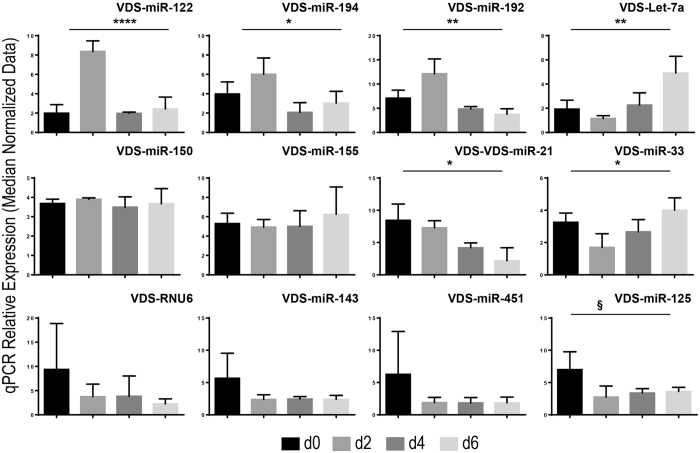
Expression of miRNAs circulating in VDS fractions of sera isolated from rats recovering from partial hepatectomy. Vesicles were precipitated from sera isolated from rats recovering PHx and RNAs were isolated from vesicles-depleted sera. qPCR data were median normalized as described in ref. 52. Data are represented as average ± standard deviation. Statistical analysis was performed by One-Way Anova (n = 3 within each group). *P ≤ 0.05; **P ≤ 0.01; ***P ≤ 0.0001; ^§^, indicate that the P value is included between: 0.05 < P ≤ 0.06.

RNAs were isolated from vesicles-depleted sera and miRNA expression measured by using miQPCR (Fig. 7). It was found that the expression of miR-122 (P = 0.0001), miR-192 (P = 0.004), miR-194 (P = 0.03)] and Let-7a (P = 0.006) followed the same pattern as observed for vesicles-associated miRNAs (top rows in Figs 6 and 7), suggesting that these miRNAs do undergo comparable modulation in both fractions. However, the expression of miRNAs associated to infection and inflammation (i.e., miR-150 and miR-155) is unchanged in the VDS fraction (Fig. 7, middle row), while the expression of regeneration-associated miRNAs [i.e., miR-21 (P = 0.01) and miR-33 (P = 0.02)] is significantly reduced in the VDS RNAs. Other small non-coding RNAs included in the panel [i.e., RNU6, miR-143, miR-451 and miR-125 (close to significance, P = 0.057)] shows a constant expression throughout 2, 4 and 6 days after PHx and a trend of downr-egulation compared to the expression measured at day 0 (See Fig. 7, lower row).

**Figure 8:**
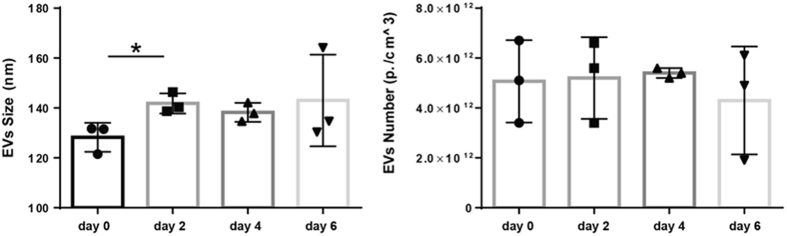
NTA analysis of EVs circulating in sera of rats recovering from partial hepatectomy. Sera isolated from rats recovering from PHx were subjected to centrifugation (20000 rcf/20 min). EVs present in the clarified supernatant were measured by using NTA. Data are represented as average ± standard deviation. Statistical analysis was performed by unpaired T-test (day 0; n = 3) versus individual regeneration groups (day 2, 4 and 6 after PHx; n = 3 within each group). *P ≤ 0.05.

Overall, a significant regulation in the expression level of miRNAs associated to both the VEP and VDS fractions 2, 4 and 6 days after PHx was identified (Figs 6 and 7). Importantly, while the expression of certain miRNAs was found to overlap across the two different populations (i.e., miR-122, miR-192 and Let-7a) the expression of other miRNAs was found to be differentially regulated. Specifically, miR-194 was found significantly up-regulated only in the VDS fractions. On the other hand, miR-150 and miR-155 were found up-regulated only in the VEP fractions, while miR-21 and miR-33 were found differentially regulated across the two populations. Although the origin and function of protein-associated miRNAs are not yet characterized, one may speculate that tissue damage in rat liver during the PHx may result in the release into the extracellular space of cellular content, including miRNA-RISC complexes, resulting in an increased amount of miRNAs associate to proteins^31^. However, the expression profiles identified in our analyses would suggest that certain miRNAs within the vesicle-depleted fraction could undergo selective regulation rather than being subjected to passive accumulation and clearance. Hence, the presented data suggest that the search for disease-relevant biomarkers should focus either on the analysis of vesicle- or protein-associated miRNAs (or both), as failing to separate these two populations (i.e., by analyzing miRNAs circulating in unfractionated serum) could lead to false negative results. Interestingly, NTA analysis of EVs circulating in the serum of rats recovering from PHx identifies that EVs size, but not the number, is significantly increased in the sera of these animals suggesting a shift toward the secretion of larger vesicles rather than an increase in the number of secreted EVs (Fig. 8).

### Cytokines modulate both the expression and the secretion of miRNAs in activated hepatic stellate cells

**Figure 9:**
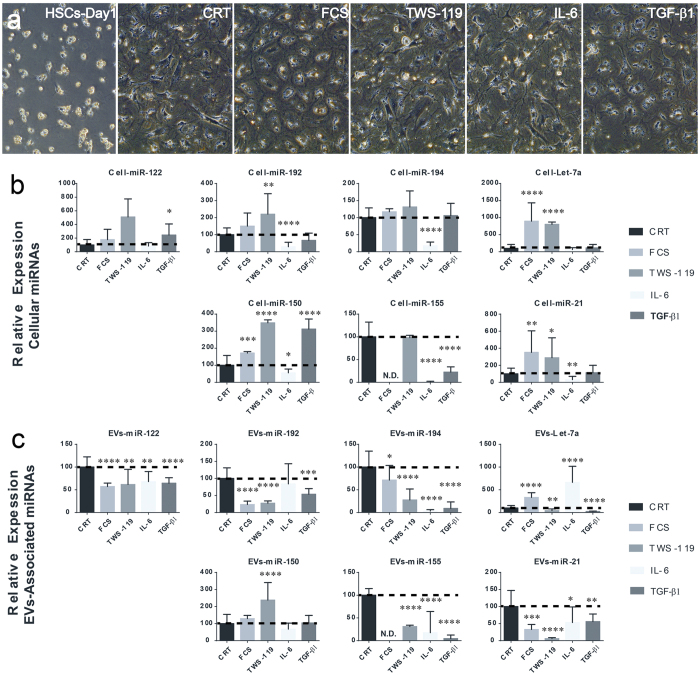
Modulation of miRNA expression and secretion in activated rat hepatic stellate cells. Following activation, rat HSCs were cultured for 24 hours in serum free medium before stimulation with either FCS or the GSK-3β inhibitor TWS-119 or cytokines (IL-6 and TGF-β1) for 72 hours. (**a**) Effect of treatments on HSCs morphology was assessed via contrast phase microscopy. Expression profiling of selected miRNAs was analyzed by miQPCR in either (**b**) cellular or (**c**) vesicles-associated RNAs. The values of unstimulated controls HSCs (CRT) were set arbitrarily to 100. Statistical analysis was performed by unpaired T-test of control group (CRT; n = 12) versus individual treatment groups (n = 9 to 12) for each miRNA. Data are represented as average ± standard deviation. *P ≤ 0.05; **P ≤ 0.01; ***P ≤ 0.0005; and ****P ≤ 0.0001. N.D. Non-detectable.

HSCs are thought to be involved liver regeneration. Following liver injury, HSCs become activate via interaction with hepatocytes^32^ contributing to tissue regeneration through the secretion of growth factors. Moreover, HSCs activation is required to arrest regeneration once the appropriate liver mass is reached^33^. In addition, HSCs may also contribute to liver regeneration by developing into progenitor-like cells^34^. In order to evaluate HSCs contribution to changes in EV-associated miRNAs observed in the serum of partially hepatectomized rats, HSCs isolated from wild type rats were cultured in serum free medium or stimulated with either FCS, IL-6, TGF-β1 or with the glycogen synthase kinase-3β (GSK-3β) inhibitor TWS-119. GSK-3β inhibition in cultured HSCs mimics the activation of canonical Wnt signaling which contributes in maintaining HSCs in a quiescent state^35^. At the end of the treatment, expression of selected miRNAs was measured by using miQPCR (Fig. 9). This analysis indicates that the expression of several miRNAs is regulated in response to cytokine administration. Interestingly, the liver specific miR-122 was found to be significantly up-regulated in activated HSCs following incubation with TGF-β1.

It was previously shown that the response to PHx is impaired in IL-6 deficient mice^36^. We now show that administration of IL-6 to activated HSCs significantly down-regulates the level of several EV-associated miRNAs, including miR-192, -194, -150, -155 and -21 (Fig. 9b). Both the molecular mechanism (i.e., whether the down-regulation of these miRNAs is directly mediated by the JAK/STAT signaling pathway) and the functional relevance (i.e., whether the down-regulation of these miRNAs in HSCs plays a roles in liver regeneration) of the observed changes remains to be determined.

Overall, we observed that the expression of cellular miRNAs shows a trend towards the up-regulation (Fig. 9b), suggesting that the activity of extracellular signals on HSCs may induce miRNA-mediated repression of miRNA-target genes. On the other hand, levels of EV-associated miRNAs secreted by HSCs were mostly down-regulated by the treatments (Fig. 9c).

## Discussion

This work presents a series of results on the isolation and characterization of cell free circulating miRNAs associated to both vesicles and Argonaute proteins. One of the major challenges towards an improved prevention and treatment of human diseases is the identification of appropriate disease-associated markers. The recent discoveries that miRNAs circulate in blood associated to vesicles has opened a new avenue for the search of disease-relevant biomarkers^1^,^2^,^3^,^4^,^5^,^6^,^7^,^8^. A growing number of publications identified significant correlations between the levels of vesicles and extracellular miRNAs in diverse human pathologies^1^,^2^,^3^,^4^,^5^,^6^,^7^,^8^. Reason for this is that, the composition as well as the relative amount of vesicles and miRNAs circulating in body fluids are expected to be modulated in a disease-specific fashion^37^,^38^. Experimental evidence indicates that extracellular vesicles play a role in inter-cellular communication and that their cargo, which includes proteins, lipids, mRNA and miRNAs, can modulate the behavior of target cell^22^,^39^,^40^,^41^. To date, two distinct populations of cell-free miRNAs have been found circulating in blood, vesicles- and protein-associated miRNAs^18^. To date, no biological function has been assigned to protein-associated miRNAs. Through this study, novel insights into methodological approaches for EVs analysis have been presented. First, we show that NTA can efficiently characterize circulating vesicles. By using NTA we could show that TEI recovers vesicles from rat serum with higher efficiency compared to ultracentrifugation (Fig. 1). Noticeably, a known shortcoming of TEI-precipitation is that this method co-precipitates lipoproteins^21^, which are known to transport miRNAs^17^. However, as the aim of this study was to assess any difference between miRNAs present in the VEP and VDS fractions of the serum rather than functionally characterize a specific group of vesicles, we concluded that the use of TEI should not affect the experimental outcome. Second, by using live imaging we show that vesicles isolated with TEI are functional and are captured and internalized by primary hepatocytes in culture (Fig. 2). Third, data presented in this study shows that serum pre-clearing steps (i.e., for removal of cellular debris) may affects EVs number (Fig. 3b) but not the size. We propose that by increasing the centrifugal force the amount of EVs that become ‘captured’ by Eppendorf’s plastic increases exponentially. Hence, the observed loss in EVs would be a random process affecting EVs of all sizes. Hence, it is paramount for all specimens to be subjected to the same identical pre-clearing procedure. Fourth, we show that serum filtration is ‘not strictly necessary’ as it fails to enrich rat serum in smaller size vesicles (Fig. 3c,d). Importantly, filtration is a commonly described approach for enriching solutions in smaller size vesicles (i.e., exosomes). However, following filtration of rat sera through 200 or 450 nm filters no effect of filtration on both vesicles distribution (Fig. 3c) and size (Fig. 3d) was observed. Fifth, we show that filtration does not affect the quantification of miRNAs isolated from the VEP and VDS fractions (Fig. 5).

Overwhelming experimental evidence indicates that cell-free circulating miRNAs are an important source for discovery of disease-associated biomarkers. However, there is controversy on whether cell-free miRNAs are predominantly associated to vesicles^18^ or to Argonaute proteins^42^ and whether the proportion of miRNAs included in these two populations is per se modulated in a diseased-specific manner. This is a very important question to be answered as selecting the analysis of one fraction over the other might possibly lead to incorrect conclusion. In this study (and in agreement with previous work from Arroyo *et al*.^18^) we show that based on their localization cell-free miRNAs from rat serum can be organized in three different groups (Fig. 4). Importantly, for the first time we show that both vesicular- and non-vesicular miRNAs are modulated in the serum of animals recovering from partial hepatectomy. Specifically, four of the investigated miRNAs (i.e., miR-122, miR-192, miR-194 and Let-7a) were congruently modulated in both the vesicular and the non-vesicular miRNA populations (Figs 6 and 7). We found that the expression levels of other two miRNAs (i.e., miR-150 and miR-155) were up-regulated only in the vesicular-fraction, while the expression of both miR-21 and miR-33 was found to be up-regulated in RNA isolated from vesicles enriched pellets and down-regulated in the RNA isolated from vesicles-depleted sera. It can be speculated that the observed modulation, which could be driven by either cells resident in the regenerating liver or in other compartment of the body, might contribute to coordinate the response of neighboring and distant cells in the different phases of liver regeneration. Moreover, we show that EVs size, but not the number, is significantly increased in the sera of rats recovering from PHx (Fig. 8). Taken together, these results provide new insights into the distribution of cell-free miRNAs circulating in serum. The presented data indicate that the analysis of miRNAs isolated from either the vesicular or the non-vesicular fractions of the serum might be more relevant in the context of hepatic disease compared to the profiling of cell-free miRNAs isolated from total serum.

We have recently shown that the stimulation of rat primary hepatocytes (PCs) with disease-associated cytokines including TGF-β1, Interleukin-1 alpha (IL-1α) and IL-6 affected the expression of both cellular and EV-associated miRNAs^27^. Specifically, we found that the cytokines used in this study significantly increased levels of miR-122, miR-150, miR-21, miR-192 and miR-194 associated to EVs secreted from PCs. We now show that also HSCs respond to humoral factors by modulating expression of cellular and EV-associated miRNAs. Among the regulated miRNAs, miR-150 is of great interest. miR-150 was found to suppress HSCs activation^43^, while Venugopal *et al*. have shown that expression of miRNA-150 was reduced in HSCs isolated from fibrotic livers^44^. We now found that miR-150 is significantly up-regulated at both cellular and vesicular level in TWS-119 treated HSCs, suggesting that canonical Wnt signaling might be required to drive the expression of this miRNA.

Overall, these data suggest that the activity of cytokines on HSCs (and PCs) during PHx-induced liver regeneration may contribute to drive the changes in cell-free miRNA observed at systemic level. Future experiments will focus on the analysis and characterization of molecular pathways down- and up-stream to the regulated cellular miRNAs, as well as in assessing the functionality of EVs-associated miRNAs in respect of inter-cellular communication.

## Methods

### Ethical Statement

The ethics committee of the University of Dusseldorf and the relevant federal state authority for animal protection (Landesamt für Natur, Umwelt und Verbraucherschutz Nordrhein-Westfalen, Recklinghausen, Germany specifically approved this study (reference number 84-02.04.2011.A133). Animals received care according to the German animal welfare act. Animal experiments included in this study were carried out in compliance with the Act No 246/1992 Coll., on the protection of animals against cruelty.

### Partial hepatectomy procedure

Adult male Wistar rats (mean body weight 213 g) were obtained from the animal facility of the Heinrich Heine University (Düsseldorf, Germany). Partial hepatectomy (PHx) was performed by surgical removal of the two largest liver lobes (approximately 70% of the liver) essentially as described^45^. The liver tissue and blood serum of the rats were collected at 2, 4 and 6 days after PHx (n = 3 for each time point) and compared with rats without surgical intervention (control, n = 3).

### Isolation of vesicles circulating in rat serum for Nanoparticle Tracking Analysis

To minimize variability associated to animal health status, age and genetic background, sera isolated from several wild type animals were pooled. Hence, if not differently indicated the pooled sera isolated of wild type (unchallenged) rats were cleared by spinning at 20000 rcf for 20 min. at 4 °C. The resulting, supernatant was moved to a fresh tube and equal amount of pooled serum were either left unfiltered (NFLS) or filtered through 0.45 μm (FL450) or 0.20 μm (FL200). Next, 200 μl of either NFLS, or FL450 or FL200 were mixed with 50 μl of Total Exosome Isolation Reagent from serum (Invitrogen, Cat: 4478360) and vesicles were pelleted by centrifugation following the instructions included in the kit. Following, centrifugation, vesicles depleted supernatant were transferred to fresh tubes while the vesicles-enriched pellets were rinsed twice with 1 ml of nuclease free water and suspended in 100 μl of nuclease free water. For the isolation of vesicles via ultracentrifugation, 10 ml of pooled-filtered serum were ultracentrifuged in an Optima GX ultracentrifuge (Beckman-Coulter) for either 120 min. or 18 hours at 120000 rcf (26000 rpm in a SW40ti rotor). Parameters for optimal ultracentrifugation conditions to achieve the pelleting of vesicles of a given size where calculated according to Livshts *et al*.^46^. Following, the supernatant were moved to fresh tubes and the pellets were rinsed twice in nuclease free water and dissolved in 550 μl of nuclease free water.

### RNA isolation from vesicles and from vesicles-depleted serum

For analysis of miRNA expression in vesicles, vesicles-enriched pellets from of 200 μl of either NFLS, FL450 or FL200 were dissolved in 500 μl of Qiazol (Qiagen, Cat: 79306) and RNA isolated following the protocols provided by the vendors with the following modification. After the separation of the water and organic phases, phenol and organic carry-over were removed from the water phases by extraction with volume - volume of chloroform and RNAs isolated with the miRNeasy mini kit (Qiagen, Cat: 217004) and eluted in 40 μl of nuclease free water. To enable the analysis of miRNAs in vesicles-depleted serum the vesicles-depleted supernatants resulting from the vesicles isolation were dissolved in 500 μl of Qiazol (Qiagen, Cat: 79306) and RNA was isolated with miRNeasy mini kit (Qiagen, Cat: 217004) as described above and eluted in 40 μl of nuclease free water. For analysis of miRNAs circulating in the serum of partially hepatectomized rats, 100 μl of serum isolated from control and treatment rats was individually centrifuged at 20000 rcf for 20 min. at 4 °C. Following vesicles were pelleted from the sera by using Total Exosome Isolation Reagent from serum (Invitrogen, Cat: 4478360) as described above. Next RNAs were extracted (see above) from vesicles-enriched pellets and vesicles-depleted supernatant and miRNAs expression was measured by miQPCR.

### Nanoparticle Tracking Analysis (NTA) of vesicles circulating in rat serum

Nanoparticle tracking analysis was performed with ZetaView multi parameter particle tracking analyzer (Particle Metrix, Germany) to measure the size distribution of vesicles. The ZetaView determines the size of vesicles based on Brownian motion and this principle is used for analysis of nanometer-sized particles^24^,^47^. Before measurements, accuracy of the ZetaView accuracy was evaluated by measuring nanoparticle with a diameter of 125 nm. Before recordings, camera focus was adjusted to make the particles appear as sharp dots, whilst the sample expected to encompass the highest vesicle number was used to set the camera sensitivity, sensitivity that was kept constant for the following measurements. Samples were diluted in ddH2O to achieve a particle count in the range of 1–9*10^7^/ml (or 100 to 300 vesicles per visual field). Using the script control function, five 30- or 60-second videos for each sample were recorded, incorporating a sample advance and a 5-second delay between each recording.

### RNA isolation, quantification and cDNA synthesis

Previous reports indicate that the concentration of cell-free RNA circulating in blood is low, in the range between 1 and 150 ng/ml^25^,^26^, suggesting that the amount of RNA that can be recovered from 200 μl of serum might be below the detection limit of quantification methods. Indeed, we were unable to quantify RNAs by using either photometric (Nanodrop) or fluorimetric (Qubit) quantification. However, as the number of cell-free circulating vesicles (and their content) is considered to be a measure of the physiological state of the organism, cDNA synthesis was carried out by using volume of elute material instead of ng of RNA (as described in ref. 27).

### Analysis of miRNA expression by using miQPCR

miRNA expression profiling by qPCR was performed by using the miQPCR method^27^,^48^. miQPCR, performs the universal reverse transcription of all the miRNAs contained in the RNA sample enabling the analysis of up to 100 individual miRNAs from every synthesized cDNA. For each analysis miRNAs contained in 4 μl of eluted RNA (or 10% of miRNeasy eluted material) were universally elongated according to the miQPCR protocol^27^ and reverse transcribed using PrimeScript (Takara, Cat: 2680A) following the supplier’s instructions. cDNAs were diluted 1:10 to 200 μl with nuclease free water and 4 μl of diluted cDNA were used in individual qPCR assays with miRNA-specific and universal primer 2.5 μM (named Upm2A, see Table 1). qPCR reactions are carried out on a Viia 7 Real-Time PCR system (Applied Biosystem), whereas amplicons were detected using SYBR Green I (GO-Taq PCR Master mix, Promega, Cat: A6002). Final volume of each assay was 15 μl. The temperature profile was: Hot start at 95 °C for 2 min. followed by 50 cycles of denaturation at 95 °C for 15 sec, annealing and elongation at 60 °C for 35 sec. Expected products were validated by recording melting curves from 65 °C to 95 °C in 0.5 °C intervals. Sequences of the miQPCR primers designed to amplify miRNAs included in this study are listed in Table 1. Data analysis was carried out by using qBase^49^. Suitable reference genes were identified by using geNorm^50^ and samples were normalized by using ΔΔCt^51^. For the experiments for which no suitable reference genes was identified, qPCR data were median normalized from within qBase by using a microarray-like approach as described by Mestdagh *et al*.^52^. Statistical analyses were performed either by using unpaired T-test of control group versus individual treatments or, when comparing more than two groups, One-Way Anova. Statistical analyses were carried out by using Prism 6 (GraphPad Software). Prism generated graphs were arranged in panels by using Adobe Illustrator CS6.

### Rat primary hepatocyte preparation and vesicle uptake

Primary hepatocytes were isolated from male Wistar rats (150–200 g) essentially as described in ref. 53. In brief, hepatocytes were isolated after serial perfusion of rat liver by Hanks’ balanced salt solution (HBSS, Sigma, Cat: H6648) and collagenase CLS type II solution (50 mg/150 ml, Biochrom, Cat: C2-22). The collagenase was dissolved in HBSS (Sigma Cat: H8264) supplemented with albumin fraction V (3 g/150 ml, Roth, Cat: CP84.1) and applied by circulated perfusion for 17–20 min. at 37 °C. After sufficient digestion, a pair of tweezers was used to disrupt the liver tissue and the resulting cell suspension was centrifuged three times at 44 rcf for 3 min. to further remove non-parenchymal cells. The hepatocyte pellet was suspended in culture medium (Williams’ E medium, Millipore, Cat: F1115) supplemented with 10% (v/v) fetal calf serum (FCS superior, Millipore, Cat: S0615), 2 mM L-glutamine (Gibco, Cat: 25030), 0.6 μg/ml insulin (Sigma, Cat: I05116), 100 nM dexamethasone in DMSO (Sigma, Cat: D8893) and 1% (v/v) penicillin-streptomycin-amphotericin B solution (Gibco, Cat: 15240–062). About 200,000 hepatocytes per well were seeded on 35 mm glass bottom dishes (Greiner Bio-One, Frickenhausen, Germany, Cat: 6780) pre-coated by collagen type I (Sigma, Cat: C3867, 6–10 mg/cm^2^). The adherent hepatocytes received new medium after 3 hours of culture at 37 °C and 5% CO_2_. Primary hepatocytes were allowed to recover for 1 hour and then stimulated with 1.5*10^12^ of either labeled or unlabeled vesicles isolated from whole rat sera as described above. The cells were incubated for 18 hours at 37 °C, 5% CO_2_ as well as saturated humidity using an onstage incubation chamber placed on an inverted microscope suitable for live cell imaging (EVOS FL Auto, Thermo Fisher Scientific). Identical settings for the excitation at 470/22 nm and detection of emitted fluorescence light at 510/42 nm were used for labeled or unlabeled vesicles during live cell imaging. Unlabeled vesicles served as controls to exclude unspecific autofluorescence of primary hepatocytes.

### Hepatic stellate cell preparation and culture

Vitamin-A-storing stellate cells were isolated from Wistar rats (>500 g) by enzymatic digestion of the liver. The non-parenchymal cell fraction was separated from hepatocytes by centrifugation (44 *g*) and stellate cells were subsequently enriched by density gradient centrifugation (8% Nycodenz, Cat:1002424, Nycomed Pharma, Oslo, Norway) as described in ref. 54. Isolated stellate cells were cultured in Dulbecco’s Modified Eagle Medium (DMEM, Cat:41966-029, Gibco) supplemented with 10% (v/v) FCS (Biochrom) and 1% (v/v) penicillin-streptomycin-amphotericin B solution (Gibco, Cat: 15240–062) or under serum-free conditions using DMEM supplemented with 1% (v/v) insulin-transferrin-sodium selenite solution containing oleic acid/linolic acid-albumin from bovine serum (ITS+3, Cat:LI2771, Sigma-Aldrich) and 1% (v/v) penicillin-streptomycin-amphotericin B solution. The serum free medium was used after attachment of freshly isolated hepatic stellate cells in DMEM/FCS at the second day of culture, when the cells were treated with growth factors or cytokines.

## Additional Information

**How to cite this article**: Castoldi, M. *et al*. Isolation and characterization of vesicular and non-vesicular microRNAs circulating in sera of partially hepatectomized rats. *Sci. Rep.* **6**, 31869; doi: 10.1038/srep31869 (2016).

## Figures and Tables

**Table 1:** Sequences of primers used for measuring miRNA expression by miQPCR.

Name	Sequences 5' > 3'
rno-Let-7a	**AGGTAGTAGGTTGTATAGTTG**
rno-miR-122	**GAGTGTGACAATGGTGTTTGG**
rno-miR-125	**TGAGGTTCTTGGGAGCCG**
rno-miR-142-3p	**TGTAGTGTTTCCTACTTTATGGAG**
rno-miR-143	**AGATGAAGCACTGTAGCTCAG**
rno-miR-150	**CCAACCCTTGTACCAGTGG**
rno-miR-155	**TTAATGCTAATTGTGATAGGGGTG**
rno-miR-15a	**TAGCAGCACATAATGGTTTGTGG**
rno-miR-15b	**TAGCAGCACATCATGGTTTACAG**
rno-miR-16	**CAGCACGTAAATATTGGCGG**
rno-miR-18a	**GGTGCATCTAGTGCAGATAGG**
rno-miR-192	**TGACCTATGAATTGACAGCCG**
rno-miR-194	**GTAACAGCAACTCCATGTGGAG**
rno-miR-21	**TAGCTTATCAGACTGATGTTGAGG**
rno-miR-223	**TCAGTTTGTCAAATACCCCAG**
rno-miR-30d	**TCCCCGACTGGAAGG**
rno-miR-33	**TGCATTGTAGTTGCATTGCAG**
rno-miR-451	**AACCGTTACCATTACTGAGTTGG**
rno-miR-92a	**CTTGTCCCGGCCTGG**
rno-miR-98	**AGGTAGTAAGTTGTATTGTTGG**
RNU6	**GCAAGGATGACACGCAAATT**
Upm2A	**CCCAGTTATGGCCGTTTA**

Upm2A indicates the sequence of the universal primer used for qPCR, while MQ-RT indicates the sequence of the universal reverse transcription primer. miRNA-primers included in this analysis were selected with respect to their expression in the liver (miR-122^55^, miR-194^55^ and miR-192^55^), their function during infection, inflammation (miR-155^56^), or regulation of HSCs function (miR-150^43^), or their increased expression during liver regeneration (miR-33^57^, miR-21^58^). Whereas, other miRNAs and small non-coding RNAs included in the panel were selected for being ubiquitously expressed (RNU6^55^ and Let-7a^55^), for being enriched in brain (miR-143^55^,^59^) or for being either associated to hematopoiesis (miR-451^60^) or hepatoblastoma (miR-125^61^).
